# Sirtuin1 mitigates hypoxia-induced cardiomyocyte apoptosis in myocardial infarction via PHD3/HIF-1α

**DOI:** 10.1186/s10020-025-01155-z

**Published:** 2025-03-14

**Authors:** Yafen Chen, Shuyao Shan, Qiqi Xue, Yan Ren, Qihong Wu, Jiawei Chen, Ke Yang, Jiumei Cao

**Affiliations:** 1 Department of Geriatrics, Ruijin Hospital, Shanghai Jiaotong University School of Medicine, 197 Rui Jin Road II, Shanghai, 200025 People’s Republic of China; 2 Department of Cardiovascular Medicine, Ruijin Hospital, Shanghai Jiaotong University School of Medicine, 197 Rui Jin Road II, Shanghai, 200025 People’s Republic of China; 3 Shanghai Institute of Cardiovascular Diseases, Zhongshan Hospital, Fudan University, Shanghai, China; 4 Department of Cardiology, Shanghai Ninth People’S Hospital, Shanghai Jiaotong University School of Medicine, 639 Zhizaoju Road, Shanghai, 200011 People’s Republic of China

**Keywords:** Acute myocardial infarction, Apoptosis, Hypoxia, Sirt1, Phd3, Hif-1α

## Abstract

**Background:**

Acute myocardial infarction (AMI) is a leading cause of mortality, characterized by myocardial ischemia that induces cardiomyocyte apoptosis and subsequent cardiac dysfunction. Sirtuin 1 (Sirt1) has emerged as a key regulator of cell survival and apoptosis, particularly under hypoxic conditions.

**Methods:**

An AMI animal model was established via ligation of the left anterior descending (LAD) coronary artery. Gene expression in the infarcted region was evaluated at various time points. Sirt1 overexpression and control lentivirus were administered to the peri-infarct region of mice heart. After LAD ligation, assessment on myocardial infarct size, cardiac function, and cardiomyocyte apoptosis were performed. In vitro, primary mouse cardiomyocytes subjected to hypoxia were analyzed for gene expression, while interactions among Sirt1, Phd3, and Hif-1α were explored using diverse treatment approaches.

**Results:**

A significant reduction in Sirt1 and Phd3 expression, along with an increase in Hif-1α and cleaved caspase-3, was observed in a time-dependent manner post-myocardial infarction (MI). In vitro findings revealed that hypoxia decreased nuclear Sirt1 and cytoplasmic Phd3 levels while promoting a time-dependent increase in Hif-1α and cleaved caspase-3. Furthermore, Sirt1 overexpression enhanced Phd3 expression in cardiomyocytes, suppressed Hif-1α and cleaved caspase-3 levels, and alleviated hypoxia-induced cardiomyocyte apoptosis. Notably, knockdown of Phd3 negated Sirt1’s inhibitory effect on Hif-1α, whereas Hif-1α knockdown promoted Sirt1 expression. Sirt1 overexpression reduced infarct size, decreased cardiomyocyte apoptosis, and improved cardiac function.

**Conclusions:**

Sirt1 effectively reduces cardiomyocyte apoptosis and myocardial infarction size while enhancing cardiac function post-MI, primarily through the Phd3/Hif-1α signaling pathway.

**Supplementary Information:**

The online version contains supplementary material available at 10.1186/s10020-025-01155-z.

## Introduction

Acute myocardial infarction (AMI) remains the leading cause of mortality among patients with cardiovascular diseases worldwide (Anderson and Morrow 2017). The pathophysiology of AMI is primarily characterized by myocardial ischemia, where the resulting hypoxic conditions trigger cardiomyocyte apoptosis and necrosis. These cellular events are crucial in the cascade leading to extensive myocardial injury and subsequent cardiac dysfunction. Amidst the complex molecular mechanisms underlying AMI, the silent information regulator 1 (Sirt1), a member of the sirtuin family, has emerged as a significant modulator of cell survival and apoptotic pathways (McDougald et al. 2018; Wu et al. 2022). This essay explores the intricate relationship between Sirt1 and AMI, particularly focusing on its regulatory role in cardiomyocyte apoptosis under hypoxic conditions.

Sirt1 is renowned for its multifaceted role in promoting cell survival and inhibiting apoptosis (Gu et al. 2019; Zhou et al. 2018). In the context of cardiovascular diseases, Sirt1 has been implicated in various protective mechanisms (Ma and Li 2015; Prola et al. 2017). Notably, it reduces apoptosis induced by endoplasmic reticulum stress in cardiomyocytes, thereby enhancing systolic function, especially in aging murine models (Hsu et al. 2017). Furthermore, ischemia–reperfusion injury, a critical component of AMI pathology, is associated with a downregulation of Sirt1 expression. Overexpression of Sirt1 in such scenarios has been shown to boost the antioxidative capacity of cardiomyocytes, thereby reducing apoptosis and preserving cardiac function (Kuno et al. 2022). Despite these insights, the precise relationship between Sirt1 and AMI, particularly in the milieu of cardiomyocytes post-myocardial infarction (MI), remains inadequately elucidated.

Ischemia-induced hypoxia is the main trigger of cardiomyocyte apoptosis during AMI. Hypoxic conditions cause changes in the expression of various cytokines, with hypoxia-inducible factor-1α (Hif-1α) playing a central role (Choudhry and Harris 2018; McGettrick and O’Neill 2020). Elevated levels of Hif-1α have been shown to facilitate hypoxia-induced apoptosis in cardiomyocytes by modulating the expression of apoptosis-related genes such as p53 and B-cell lymphoma 2 (Bcl-2) (Malhotra et al. 2008). Further elucidating this pathway, our previous research identified that miR-10b influences cardiomyocyte apoptosis under hypoxic conditions by downregulating the PTEN/Hif-1α axis (Wu et al. 2019). Additionally, prolyl hydroxylase 3 (Phd3) has been implicated in the regulation of Hif-1α hydroxylation, thereby controlling its nuclear transport and intracellular concentrations, which in turn affects Hif-1α’s functional repertoire (Fong and Takeda 2008). However, the interplay between Sirt1 and this Hif-1α regulatory mechanism via Phd3 remains unexplored.

We hypothesize that under hypoxic conditions prevalent in AMI, Sirt1 downregulation diminishes its regulatory influence on Phd3, leading to a surge in Hif-1α levels. This Hif-1α accumulation subsequently triggers widespread cardiomyocyte apoptosis, contributing to impaired cardiac function. To investigate this hypothesis and delineate the underlying mechanisms, we employed an in vivo murine model of myocardial infarction and primary murine cardiomyocytes for mechanistic analyses. Our study aims to define the reciprocal regulatory relationship between Sirt1 and the Phd3/Hif-1α axis, thereby elucidating the role of Sirt1 in modulating cardiomyocyte apoptosis following AMI. This investigation will contribute to a more comprehensive understanding of the molecular mechanisms governing post-infarction myocardial injury and may inform the development of novel therapeutic strategies targeting Sirt1 to mitigate the devastating consequences of AMI.

## Methods

### Materials and reagents

Cell culture media and solutions, including M199 medium (Cat# 12,350,039) and fetal bovine serum (FBS, Cat# 10,099), were sourced from Thermo Fisher Scientific (MA, USA), along with TRIzol reagent for RNA extraction (Cat# 15,596,026), enhanced chemiluminescence (ECL) kits for protein detection (Cat# A38554), Masson staining kits for histological analysis, and bicinchoninic acid (BCA) protein assay kits for protein quantification (Cat# 23,227). Trypsin (0.25%, Cat# 1,001,002), Hank’s balanced salt solution (Cat# 14,175,095), and phosphate-buffered saline (PBS, Cat# 25,200,072) were obtained from Gibco (CA, USA), ensuring consistency in cell handling procedures. For enzymatic tissue digestion, type II collagenase was acquired from Worthington Biochemical Corporation (Cat# 9001-12-1, NJ, USA). Apoptotic cell death was assessed using TUNEL assay kits (Cat# 11,684,795,910) from Roche (CA, USA). The assessment of myocardial infarct size employed 2,3,5-triphenyl tetrazolium chloride (TTC, Cat# T8877) and Evans blue stain (Cat# E2129), both obtained from Sigma-Aldrich (MA, USA). Molecular analyses incorporated SYBR Green PCR Master Mix (Cat# 4,472,903) from Applied Biosystems (ABI, MA, USA), with primers for sirtuins Phd3 and β-actin designed using Primer5 software to ensure specificity and efficiency in quantitative PCR. A comprehensive panel of antibodies was used for immunodetection. Primary antibodies against Sirt1 (Cat# 9475), Hif-1α (Cat# 36,169), cleaved caspase-3 (Cat# 9661), total caspase-3 (Cat# 9662), α-actin (Cat# 6487), and β-actin (Cat# 3700) were obtained from Cell Signaling Technology (MA, USA). The Phd3 antibody (Cat# ab184714) was sourced from Abcam (UK). Secondary antibodies, including anti-rabbit (Cat# 14709s), anti-mouse (Cat# 14708s), and Alexa Fluor 555-conjugated antibodies (Cat# 4413), facilitated the detection of primary antibody binding and were also supplied by Cell Signaling Technology. For genetic manipulation, lentiviral vectors were constructed by GeneChem (Shanghai, China). This included the negative control lentiviral vector and a Sirt1-overexpressing lentiviral vector containing the cardiac-specific cTNT promoter (Cat# GSPE0156704) to drive targeted expression in cardiomyocytes. Additionally, a Hif-1α knockout lentiviral vector utilizing the CRISPR/Cas9 system (Cat# GOSL0191389) enabled precise gene editing to investigate the role of Hif-1α in cardiac function.

### Animals

Neonatal specific-pathogen-free (SPF) C57BL/6 mice aged 1–3 days were used for primary cardiomyocyte culture, while male SPF-grade C57BL/6 mice aged 6–8 weeks (body weight 18–20 g) were utilized for in vivo experiments. All mice were obtained from Beijing Vital River Laboratory Animal Products Co., Ltd. The adult mice were acclimated to the laboratory environment for one week before experimentation. Animal care and experimental procedures adhered to the principles of Replacement, Reduction, and Refinement (3Rs) to ensure ethical treatment and minimize animal use and suffering.

### Animal model of myocardial infarction and in situ lentiviral myocardial injection

In this study, we developed a mouse model of myocardial infarction (MI) to examine the effects of Sirt1 overexpression on cardiac function post-infarction. MI model was established using male SPF-grade C57BL/6 mice aged 6–8 weeks (body weight 18–20 g). Mice were anesthetized via intraperitoneal injection of sodium pentobarbital (20 mg/kg) (Wu et al. 2019; Xue et al. 2023; Zhang et al. 2024). A left thoracotomy was performed through the fourth intercostal space, and the left anterior descending (LAD) coronary artery was ligated under a stereomicroscope to induce AMI as previously described (Wu et al. 2019).

**Fig. 1:**
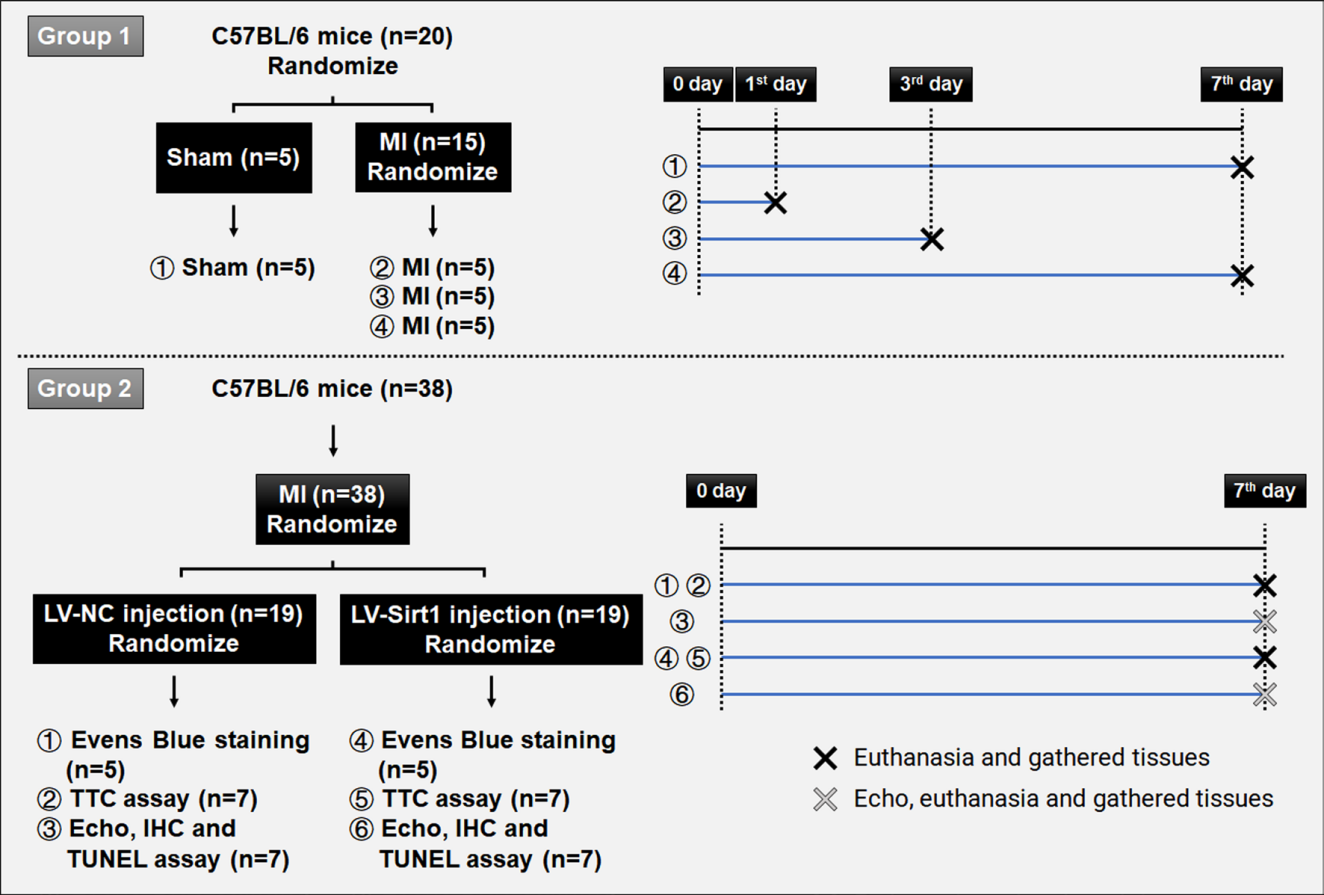
Flowchart of the animal model

To evaluate the therapeutic potential of Sirt1 overexpression, mice were divided into two groups (n = 19 per group). One group received in situ injections of a Sirt1-overexpressing lentivirus (LV-Sirt1) (Higuchi et al. 2009), while the control group received a blank lentivirus (LV-NC). Each mouse was injected with a total of 4 × 10^6^ transducing units, delivered as 1 × 10^6^ units per site at four distinct locations within the infarcted myocardial tissue near the ligation site. Figure 1 illustrates the experimental procedure used in this study. At the end of the experimental timeline, all mice were humanely euthanized using isoflurane inhalation, followed by an intraperitoneal injection of sodium pentobarbital at a dose of 30 mg/kg. The hearts were then collected for further analyses.

This study adhered to ethical standards and was approved by the Ethics Committee of Ruijin Hospital, Shanghai Jiaotong University School of Medicine (Reference number: Ruijin-YK-01).

### TTC and Evans blue staining

After perfusion with 0.90% normal saline, the hearts were sectioned into five slices along the coronal plane. TTC staining was performed following the protocol previously described (Wu et al. 2019). To quantify the myocardial infarction (MI) area, images were analyzed using Image-Pro Plus software (v6.0; Media Cybernetics). The area at risk (AAR) was identified using both Evans Blue and TTC staining methods, as detailed in prior studies (van Hout et al. 2017). AAR measurements were also conducted with Image-Pro Plus software (v6.0; Media Cybernetics, Rockville, MD, USA).

### Cardiac function analysis

Cardiac function was assessed through two-dimensional echocardiography conducted at baseline (pre-surgery) and on postoperative Days 1, 3, and 7 following myocardial infarction (MI). The evaluations were performed using a Vevo 2100 system (Fujifilm Visual Sonics) equipped with MS-400 transducers, adhering to the methods previously established (Wu et al. 2019).

### Primary mouse cardiomyocyte culture and treatment

Primary cardiomyocytes were isolated from the myocardial tissues of neonatal mice euthanized via diethyl ether inhalation using an established enzymatic digestion method (Wu et al. 2019). After isolation, the cardiomyocytes were cultured in 60 mm dishes under normoxic (21% O_2_) conditions as controls or exposed to hypoxic conditions (2% O_2_) for varying durations (1, 3, 6, 9, and 12 h).

To examine the roles of Sirt1 and Hif-1α, cells were transfected with either a Sirt1-overexpressing lentiviral vector (LV-Sirt1), a CRISPR/Cas9-mediated Hif-1α knockout vector [LV-Hif-1α (-)], or a negative control vector (LV-NC), each at a concentration of 1 × 10^5^ transducing units per culture medium. Following a 24-h incubation period to allow for transduction, the efficiency of gene transfection and the expression levels of target genes were analyzed using western blot analysis. Following transfection, cells were either maintained under normoxic conditions or subjected to hypoxia as described.

### Real-time PCR

Total RNA was isolated following the manufacturer’s instructions. All procedures, including tissue homogenization and RNA extraction, were conducted on ice to prevent RNA degradation. Briefly, 0.2 mL of chloroform was added to each sample and homogenized thoroughly. After a 2-min incubation, the samples were centrifuged at 12,000×*g* at 4 °C for 15 min using an Eppendorf 5430R microcentrifuge. The aqueous (upper) phase was carefully transferred to a new tube. Subsequently, 0.2 mL of isopropanol was added to each sample, followed by homogenization, a 10-min incubation, and centrifugation at 12,000×*g* at 4 °C for 10 min. The supernatant was discarded, and the RNA pellets were washed with 75% ethanol, vortexed briefly, and centrifuged at 7500×*g* at 4 °C for 5 min. After the supernatant was removed, the RNA pellets were air-dried for 10 min and resuspended in 15 µL of RNase-free water.

RNA concentrations were measured using the NanoDrop™ 2000 spectrophotometer (Thermo Fisher™, ND-2000). Complementary DNA (cDNA) synthesis was performed using 0.5 µg of RNA per sample with 5X PrimeScript RT Master Mix (TaKaRa, RR036A), following the manufacturer’s protocol. Specifically, 0.5 µg of RNA was transferred to a clean 8-strip 200 µL tube, and the volume was adjusted to 8 µL using RNase-free water. Next, 2 µL of 5X PrimeScript RT Master Mix was added to each sample, mixed thoroughly, and briefly centrifuged. Reverse transcription was carried out using a PCR Thermal Cycler T100™ (Bio-Rad Laboratories, 1,861,096) with the following settings: 37 °C for 15 min, followed by 85 °C for 5 s. The resulting cDNA was diluted 1:3 with RNase-free water and stored at −20 °C until further use.

Quantitative real-time PCR (qPCR) was performed using TB Green® Premix Ex Taq™ (Tli RNaseH Plus) (TaKaRa, RR420A) on the QuantStudio 5 Real-Time PCR System (Thermo Fisher Scientific, A28140), following the manufacturer’s instructions. The reaction began with a pre-denaturation step at 95 °C for 30 s (1 cycle), followed by 40 cycles of denaturation at 95 °C for 5 s and annealing/extension at 60 °C for 30–34 s. Relative mRNA expression levels were normalized to the endogenous control gene HPRT, and fold changes were calculated using the ΔΔCt method. Statistical significance was determined using either two-way ANOVA or a two-tailed Student’s t-test, with p < 0.05 considered significant.

Gene-specific primers were validated using the Primer-BLAST tool on the NCBI platform. In this process, primer sequences were entered into the “Primer Parameters” section, followed by the selection of the target database (e.g., RefSeq mRNA or Genome) and species in the specificity check section. The specificity of the primers was assessed based on the BLAST results, including gene matches, product size, and primer-template complementarity. Specific primer sequences used for amplification are detailed in Supplementary Table S1.

### Western blot analysis

Proteins were extracted from tissue samples using a lysis buffer containing a cocktail of protease inhibitors to prevent degradation. Protein concentrations were quantified, and equal amounts of protein were separated by SDS-PAGE. Proteins were then transferred onto polyvinylidene difluoride (PVDF) membranes as described previously (Wu et al. 2019). Membranes were blocked with skim milk to minimize nonspecific binding and incubated with primary antibodies against Sirt1, Hif-1α, Phd3, cleaved caspase-3, caspase-3, or β-actin, according to the manufacturers’ protocols. After primary antibody incubation, membranes were probed with appropriate horseradish peroxidase-conjugated secondary antibodies. Protein bands were visualized using an enhanced chemiluminescence (ECL) detection kit. Images were captured with a Gel Doc imaging system and analyzed quantitatively using ImageJ software.

### Immunohistochemical and histological analysis

Hearts were collected from mice (n = 7 per group) and fixed overnight in 4% paraformaldehyde at 4 °C. The fixed tissues were embedded in optimal cutting temperature (OCT) compound and cryosectioned into 6 μm thick slices. Tissue sections underwent histological examinations using hematoxylin and eosin (H&E) staining to evaluate general myocardial morphology and Masson’s trichrome staining to determine the extent of fibrosis in the infarcted regions.

### TUNEL assay and tissue immunofluorescence

Apoptotic cells in cardiac tissue sections were identified using the TUNEL assay, performed with an in-situ cell death detection kit (Roche) according to the manufacturer’s instructions. To specifically label cardiomyocytes, the sections were first incubated with an anti-α-actinin primary antibody at a 1:50 dilution. After thorough rinsing to remove unbound antibodies, the sections were incubated in the dark with an Alexa Fluor 555-conjugated secondary antibody (1:1000 dilution) to visualize α-actinin-positive cells. Nuclear counterstaining was performed using DAPI at a 1:1000 dilution. The prepared sections were examined and imaged using a Zeiss LSM 710 laser confocal microscope. The number of TUNEL-positive apoptotic cells was quantified using ZEN 2.3 software (Zeiss), enabling precise evaluation of apoptosis within the cardiac tissue.

### Cellular immunofluorescence analysis

To analyze protein expression in cultured cells, cells were fixed in 4% paraformaldehyde to preserve cellular structure and permeabilized with 0.1% Triton X-100 in PBS at room temperature to allow antibody access to intracellular targets. Non-specific binding sites were blocked with 5% horse serum at 4 °C. The cells were then incubated overnight at 4 °C with primary antibodies against Sirt1 (1:100 dilution) or Phd3 (1:250 dilution), proteins relevant to cardiovascular pathology. After primary antibody incubation, cells were washed and incubated with Alexa Fluor 555-conjugated secondary antibodies (1:200 dilution) in the dark to prevent photobleaching of the fluorophore. Nuclei were stained with DAPI (1:1000 dilution) to facilitate cellular localization and morphological analysis. Finally, the slides were mounted and visualized using the Zeiss LSM 710 laser confocal microscope, allowing high-resolution imaging of protein localization and expression patterns in the cells.

### RNA sequencing

Raw reads generated from the Illumina HiSeq2500 sequencer were demultiplexed using bcl2fastq version 2.19.0. Differential expression analysis was performed using DESeq package 1.38.3 with a p value threshold of 0.05 within R version 4.2.3. Heatmaps were generated using heatmap package 1.0.12 with the Log transformed expression values. Barplot were generated using ggplot2 package 3.5.0.

### Statistical analysis

All statistical analyses were conducted using SPSS version 20.0 (IBM Corp). Data were obtained from at least three independent experiments and are expressed as mean ± standard deviation (SD). The normality of data distributions was initially evaluated using the Kolmogorov–Smirnov test. For datasets following a normal distribution, a one-way analysis of variance (ANOVA) was performed. When the data did not conform to a normal distribution, the Kruskal–Wallis test was applied. A *p*-value of less than 0.05 was considered statistically significant.

## Results

### Expression dynamics of Sirt1, Hif-1α and cleaved caspase-3/caspase-3 post-AMI

**Fig. 2:**
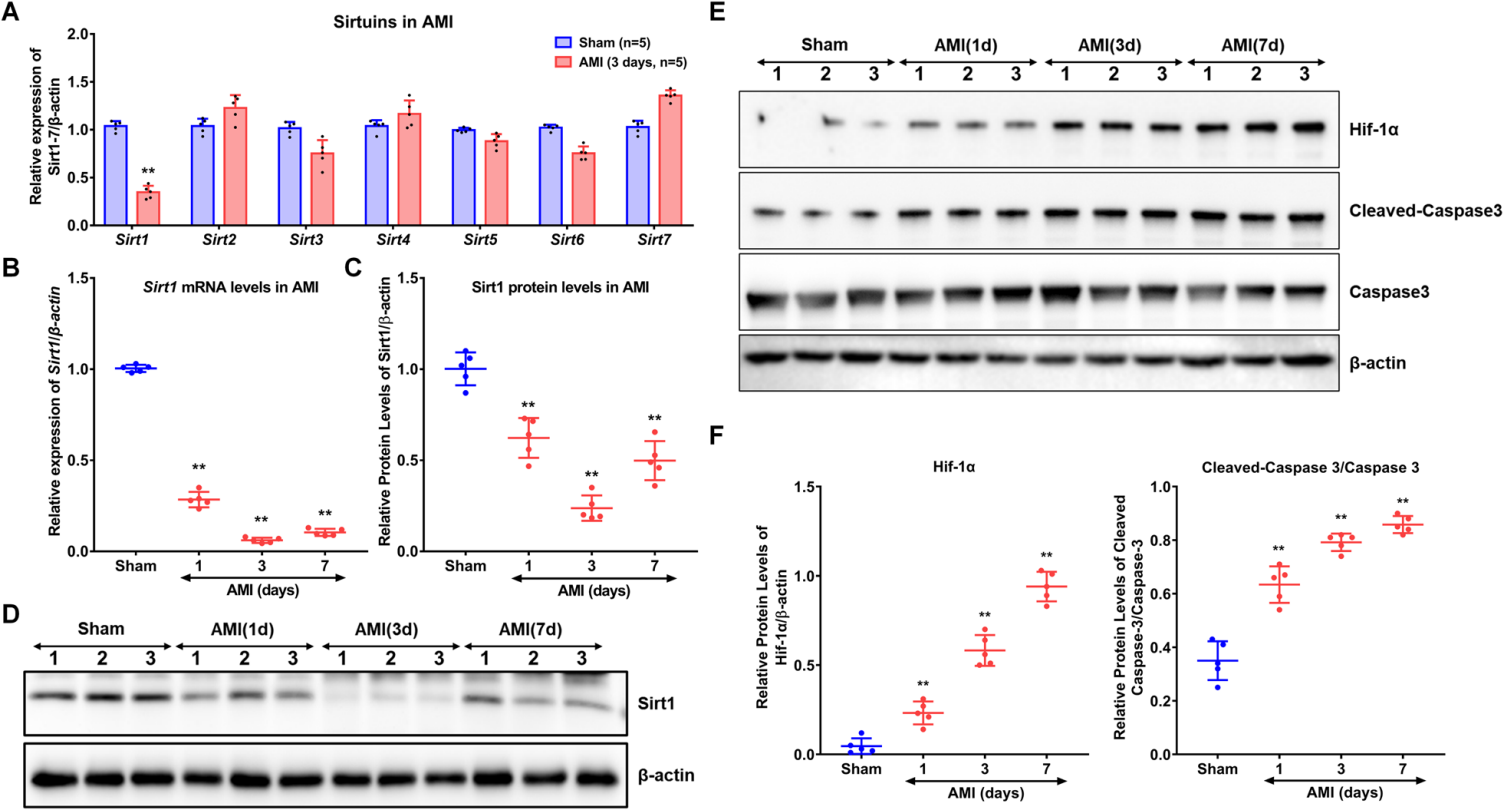
Reduced Sirt1 expression in Infarcted Hearts. **A** C57BL/6 mice underwent left anterior descending (LAD) artery ligation to induce myocardial infarction (MI). Three days post-MI, mRNA levels of the sirtuin family were measured using RT-qPCR, with the sham group as the control and β-actin as the reference gene for normalization. **B**–**D** The mRNA and protein expression levels of Sirt1 in the infarcted region were analyzed at different time points (1, 3, and 7 days) post-MI using RT-qPCR and western blotting, respectively. β-Actin was used as an internal control to calculate the relative expression of Sirt1 (n = 5, expressed as mean ± SD; compared to the sham group using repeated-measures ANOVA followed by the Dunnett multiple comparison test, **P < 0.01). **E**, **F** Furthermore, the protein levels of Hif-1α and cleaved caspase-3/caspase-3 were quantified via western blotting at the same time points post-MI, with β-actin serving as an internal control. The relative expression levels of Hif-1α and cleaved caspase-3/caspase-3 were also calculated (n = 5; results expressed as mean ± SD; compared to the sham group using repeated-measures ANOVA followed by the Dunnett multiple comparison test, **P < 0.01)

RNA sequencing (RNA-seq) was performed on RNA from cardiomyocytes of neonatal mice exposed to either normoxia or hypoxia condition. We observed differences in the expression of sirtuin family genes between the hypoxic and normoxic groups, with Sirt1 exhibiting the largest fold change and showing a significant decrease (P < 0.01) (Supplementary Fig. 1). Consistently, following acute myocardial infarction (AMI), we observed a marked decrease in Sirt1 expression, accompanied by an increase in Hif-1α and the cleaved caspase-3/caspase-3 ratio within the infarcted myocardial regions. To investigate the effects of AMI on Sirt1 expression, we conducted quantitative RT-PCR analyses of sirtuin family mRNA levels in the myocardium three days post-AMI. Among these, Sirt1 mRNA exhibited the most significant reduction compared to the sham-operated group (Sirt1 relative expression: sham = 1.04 ± 0.05 vs. AMI = 0.35 ± 0.07, P < 0.01, Fig. 2A). Subsequent RT-PCR and Western blot analyses revealed a significant, time-dependent decline in both mRNA and protein levels of Sirt1 in the infarcted regions, with the steepest reduction observed on day three post-AMI (relative Sirt1 mRNA: sham = 1.00 ± 0.01 vs. AMI day 3 = 0.06 ± 0.01, P < 0.01; relative Sirt1 protein: sham = 1.00 ± 0.09 vs. AMI day 3 = 0.24 ± 0.07, P < 0.01, Fig. 2B–D). Concurrently, there was a significant, time-dependent increase in Hif-1α expression and the cleaved caspase-3/caspase-3 ratio, reflecting heightened hypoxic stress and apoptosis in the infarcted myocardium (Fig. 2E and F). These molecular changes, including elevated apoptotic markers, highlight increased cardiomyocyte apoptosis in the infarcted tissue.

### Hypoxia inhibits Sirt1 expression while promoting Hif-1α and cleaved caspase-3 in cardiomyocytes

**Fig. 3:**
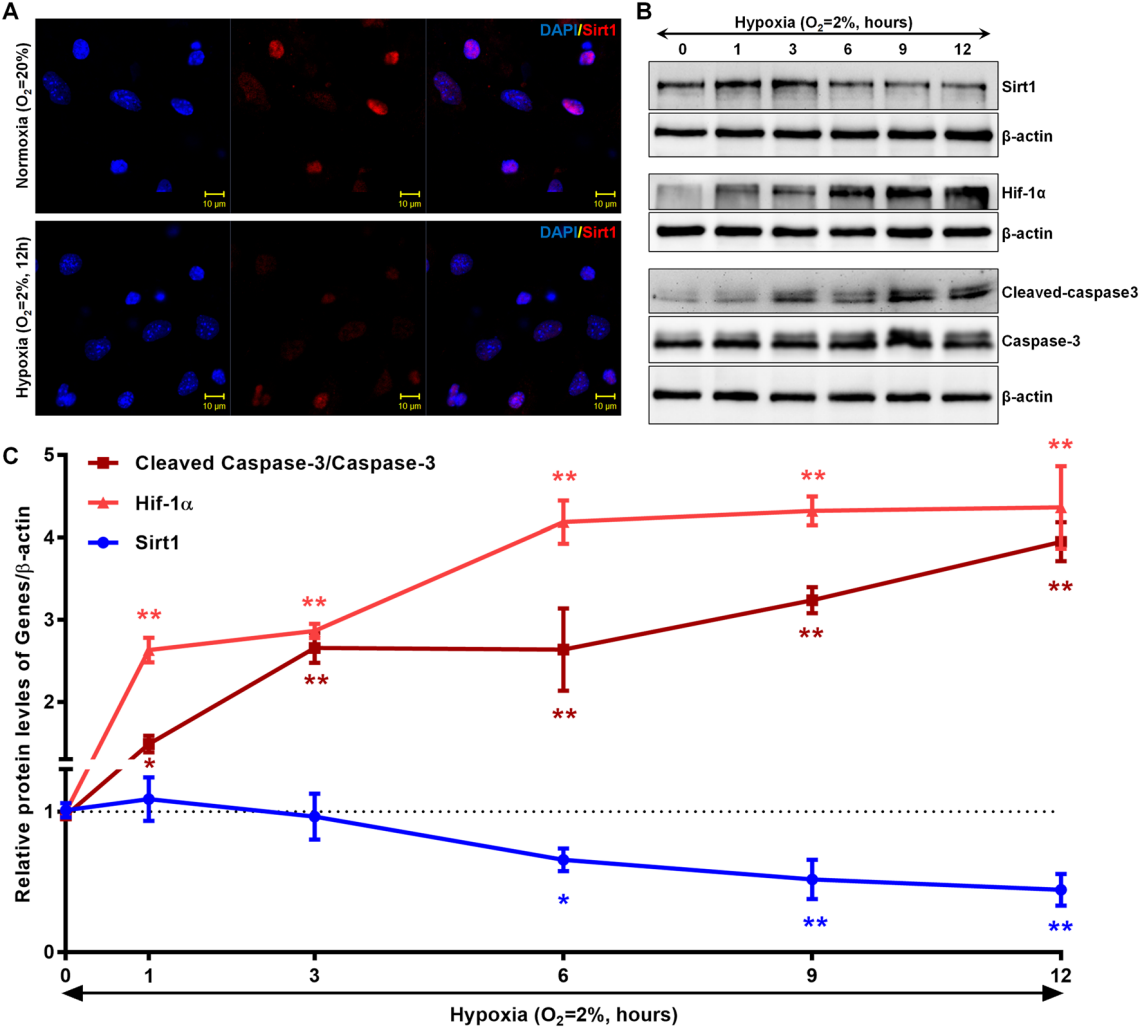
Impact of Hypoxia on Sirt1, Hif-1α, and Cleaved Caspase-3 Expression in Cardiomyocytes. **A** Cardiomyocytes were exposed to hypoxic conditions for 12 h, followed by immunofluorescence analysis to evaluate Sirt1 expression. Red fluorescence indicates Sirt1, while blue fluorescence marks cell nuclei stained with DAPI (n = 3). **B** In another set of experiments, cardiomyocytes were subjected to hypoxia for varying durations (1, 3, 6, 9, and 12 h). Protein expression levels of Sirt1, Hif-1α, and cleaved caspase-3 were then analyzed using Western blotting (n = 3). The normoxia group served as the control, with β-actin used as the internal reference. **C** Quantitative changes in Sirt1, Hif-1α, and cleaved caspase-3 relative to total caspase-3 are shown, with the red curve, dark red line, and blue line representing Hif-1α, cleaved caspase-3/caspase-3, and Sirt1 expression levels, respectively. Statistical analysis was conducted using repeated-measures ANOVA with Dunnett’s multiple comparison test, showing significance at *P < 0.05 and **P < 0.01 compared to the normoxia group (n = 3)

To investigate the regulatory effects of hypoxic conditions on Sirt1 expression, we cultured primary mouse cardiomyocytes in vitro under hypoxic conditions (O_2_ = 2%) for 12 h and subsequently analyzed Sirt1 expression levels. Our findings revealed that Sirt1 was predominantly localized in the nuclei of cardiomyocytes, and its expression significantly decreased after 12 h of hypoxic exposure (Fig. 3A). Furthermore, we examined the protein expression of Sirt1, Hif-1α, and cleaved caspase-3 at various time points during hypoxia using Western blot analysis. Compared to normoxic controls (O_2_ = 20%), we observed a marked reduction in Sirt1 protein levels in hypoxic cardiomyocytes, along with an increase in Hif-1α and cleaved caspase-3 expression (Fig. 3B). Notably, as the duration of hypoxia increased, Sirt1 levels continued to decline while Hif-1α and the ratio of cleaved caspase-3 to total caspase-3 rose. Moreover, statistical analysis revealed significant negative correlations between Sirt1 expression and the levels of both Hif-1α and cleaved caspase-3/caspase-3 in cardiomyocytes exposed to hypoxic conditions (Fig. 3C). These results highlight the intricate interplay between Sirt1 regulation and hypoxia-induced cellular stress responses in cardiomyocytes.

### Hypoxia suppressed Phd3 expression

**Fig. 4:**
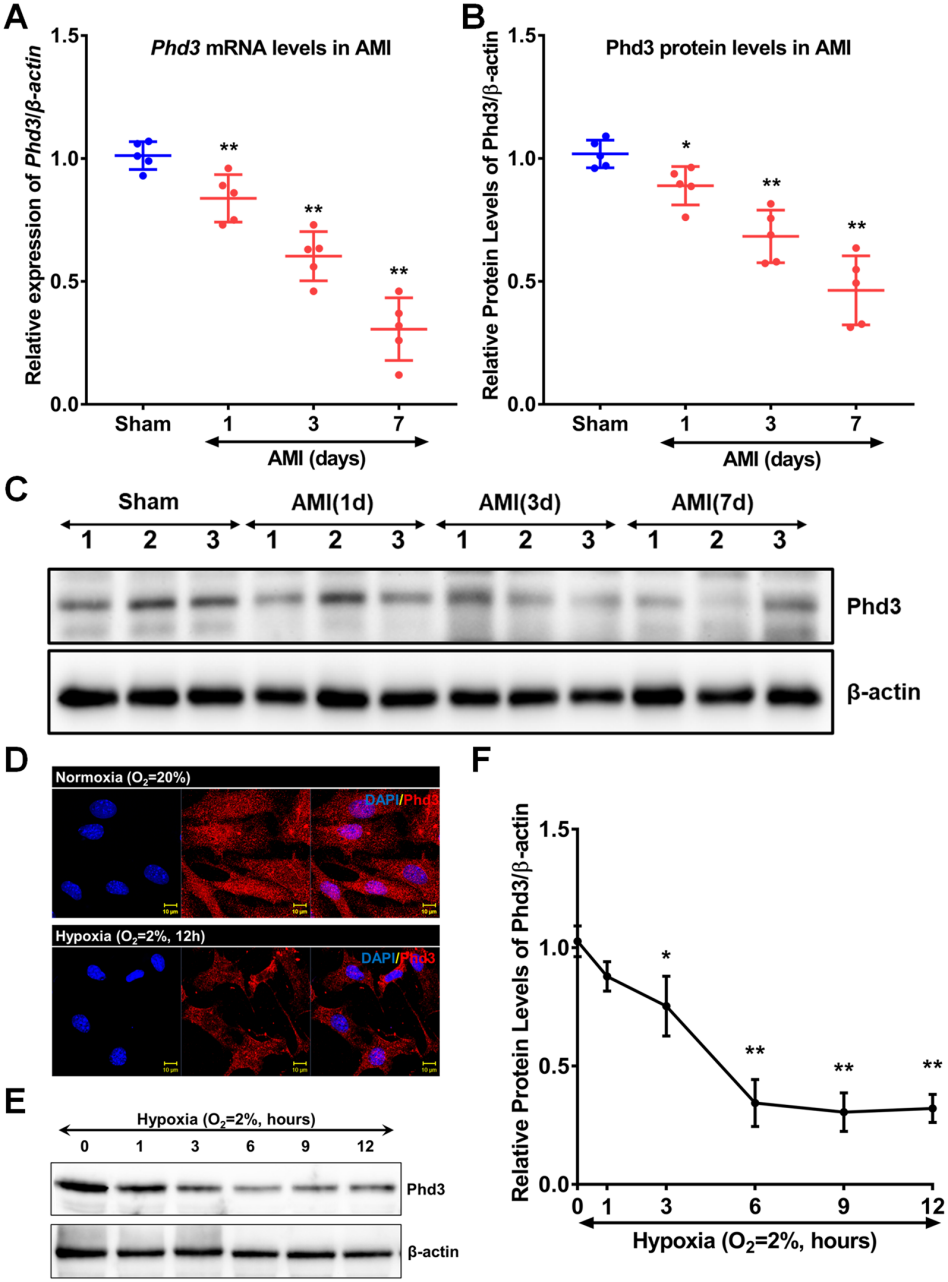
Influence of Acute Myocardial Infarction and Hypoxia on Phd3 Expression. **A** Phd3 mRNA expression in the myocardial infarction region was assessed using reverse transcription polymerase chain reaction (RT-PCR), with β-actin as the internal control for relative quantification. **B**, **C** Western blot analyses were performed to evaluate Phd3 protein levels in the MI region, using β-actin as the internal reference for relative expression (n = 5; comparisons made against the sham group; *P < 0.05, **P < 0.01, using repeated-measures ANOVA followed by Dunnett’s multiple comparison test). **D** Cardiomyocytes subjected to 12 h of hypoxia were analyzed for Phd3 expression via immunofluorescence, where red fluorescence indicates Phd3 and blue fluorescence represents nuclei (DAPI) (n = 3). **E**, **F** Cardiomyocytes were exposed to hypoxia for varying durations (1, 3, 6, 9, and 12 h) before harvest; subsequent western blot analysis quantified Phd3 expression, with β-actin as the internal control (n = 3; *P < 0.05, **P < 0.01 compared to the normoxia group, using repeated-measures ANOVA followed by Dunnett’s multiple comparison test)

To evaluate the expression dynamics of Phd3, we utilized RT-PCR and western blot analyses to measure mRNA and protein levels in infarcted cardiac tissues. Our results indicated a significant reduction in both Phd3 mRNA and protein expression in the AMI group compared to the sham-operated group, with the most substantial decrease observed on Day 7 post-myocardial infarction. Specifically, the relative expression levels of Phd3 mRNA were 1.01 ± 0.06 in the sham group and 0.31 ± 0.13 in the AMI group (P < 0.01). Similarly, Phd3 protein levels declined from 1.02 ± 0.06 in the sham group to 0.46 ± 0.14 in the AMI group (P < 0.01, Fig. 4A–C). Phd3 is primarily expressed in cardiomyocytes, but its expression is significantly lower in the AMI group compared to the Sham group (Supplementary Fig. 2). To further investigate the effects of hypoxia, we performed immunofluorescence and western blot analyses on mouse cardiomyocytes exposed to in vitro hypoxic conditions. These experiments revealed that Phd3 expression was markedly reduced in hypoxic cardiomyocytes compared to those under normoxic conditions after 12 h of exposure (Fig. 4D). Quantitatively, the Phd3 protein level in the normoxia group was 1.03 ± 0.07, whereas it decreased to 0.32 ± 0.06 in the hypoxia group (P < 0.01, Fig. 4E and F). Collectively, these findings underscore the suppressive effects of both AMI and hypoxia on Phd3 expression in cardiac tissues.

### Sirt1 inhibited hypoxia-induced apoptosis via upregulating Phd3 to suppress Hif-1α

**Fig. 5:**
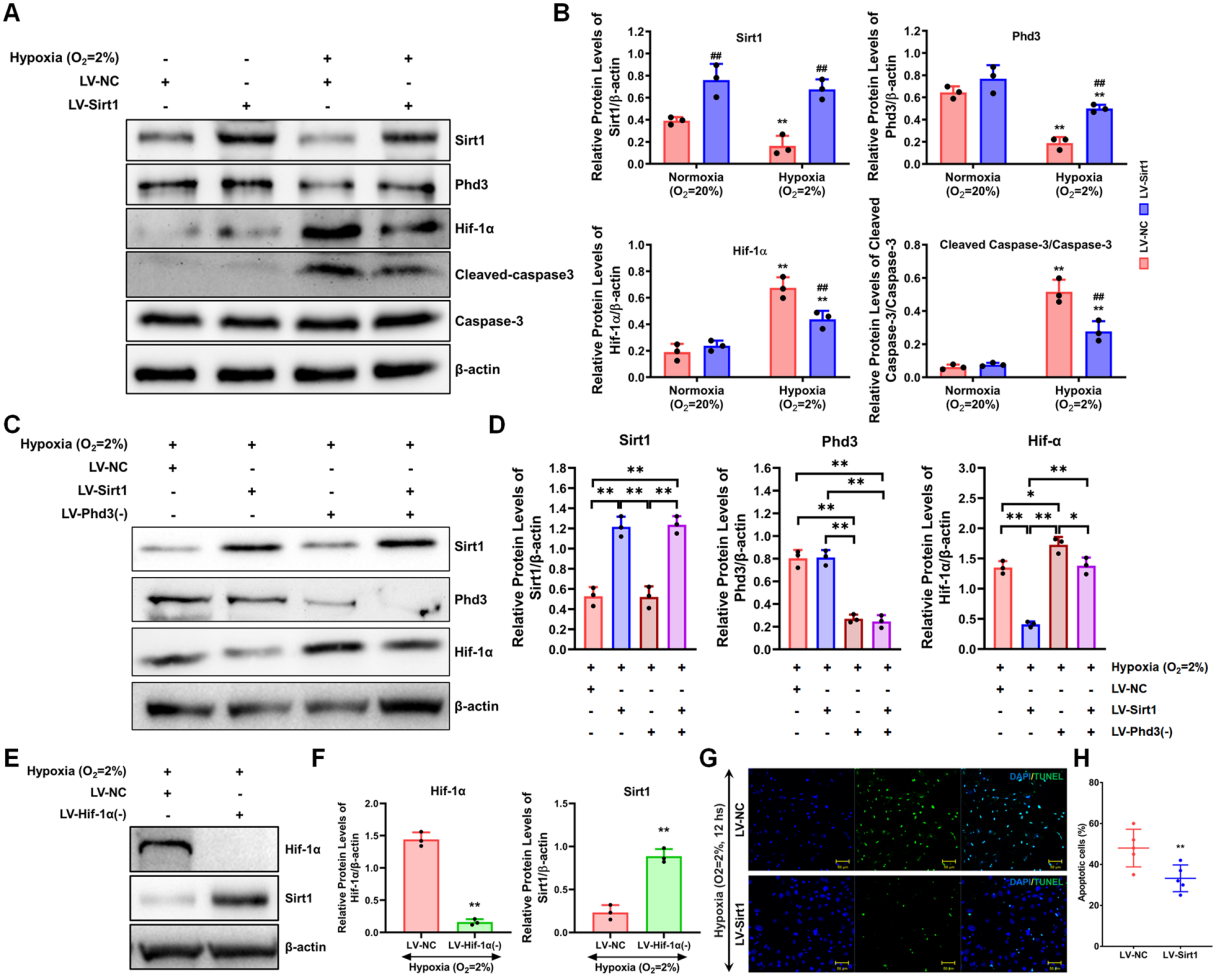
Impact of Sirt1 Overexpression on Phd3/Hif-1α Expression and Cardiomyocyte Apoptosis. **A**, **B** The expression levels of Phd3, Hif-1α, and cleaved caspase-3/caspase-3 were analyzed via western blotting, using a two-way ANOVA followed by Dunnett’s multiple comparison test and the Bonferroni posttest to determine statistical significance (n = 3). Results showed a significant difference compared to the non-hypoxic group (**P < 0.01) and the LV-NC group (##P < 0.01). **C**, **D** The effects of Sirt1 overexpression and/or Phd3 knockdown on Hif-1α expression were assessed through western blotting, with statistical analysis performed using two-way ANOVA, Dunnett’s multiple comparison test, and the Bonferroni posttest (n = 3, *P < 0.05; **P < 0.01). **E**, **F** The impact of Hif-1α knockdown on Sirt1 expression was evaluated via western blotting, with statistical significance determined using the Kruskal–Wallis test (n = 3, *P < 0.05; **P < 0.01). **G**, **H** The role of Sirt1 overexpression in regulating cardiomyocyte apoptosis was examined using the TUNEL assay. A lentiviral vector overexpressing Sirt1 was transfected into primary mouse cardiomyocytes, and apoptosis was measured following 12 h of hypoxic exposure. Blue fluorescence (DAPI) marked cell nuclei, while green fluorescence (TUNEL) indicated apoptotic cells, with results expressed as mean ± SD (n = 5, **P < 0.01 compared to the negative control [LV-NC], analyzed via the Kruskal–Wallis test)

In this study, we examined the expression profiles of Sirt1 and Phd3, which were found to be downregulated, while Hif-1α and cleaved caspase-3 were upregulated under hypoxic conditions associated with AMI. To explore the interactions among these genes, cardiomyocytes were transfected with either a negative control lentiviral vector or a lentiviral vector designed for Sirt1 overexpression, and gene expression was analyzed under both hypoxic and normoxic conditions. In the negative control group (LV-NC), hypoxia resulted in decreased Sirt1 and Phd3 expression, along with increased levels of Hif-1α and cleaved caspase-3 compared to normoxic controls. Notably, Sirt1 overexpression reversed the hypoxia-induced downregulation of Phd3 and mitigated the rise in Hif-1α and cleaved caspase-3 levels, as shown in Fig. 5A and B, which also document the efficiency of Sirt1 overexpression. Furthermore, additional experiments involving Sirt1 overexpression and Phd3 knockdown during hypoxia revealed that Phd3 knockdown enhanced Hif-1α expression, thereby reducing the protective effect of Sirt1 overexpression against Hif-1α elevation (Fig. 5C and D, with knockdown efficiency shown in Fig. 5C). Moreover, Hif-1α knockout was found to upregulate Sirt1 expression under hypoxic conditions (Fig. 5E and F; knockdown efficiency of Hif-1α shown in Fig. 5E). Importantly, Sirt1 overexpression significantly reduced hypoxia-induced apoptosis in cardiomyocytes after 12 h of exposure, with a marked difference observed between the LV-NC group (48.00 ± 9.19%) and the LV-Sirt1 group (33.20 ± 6.53%, **P < 0.01, Fig. 5G and H). Taken together, these results demonstrate that hypoxia ignites a regulatory loop involving Sirt1/Phd3/Hif-1α, ultimately resulting in apoptotic cascade in cardiomyocyte.

### Overexpression of Sirt1 exhibited cardioprotective effects in post-MI murine models

**Fig. 6:**
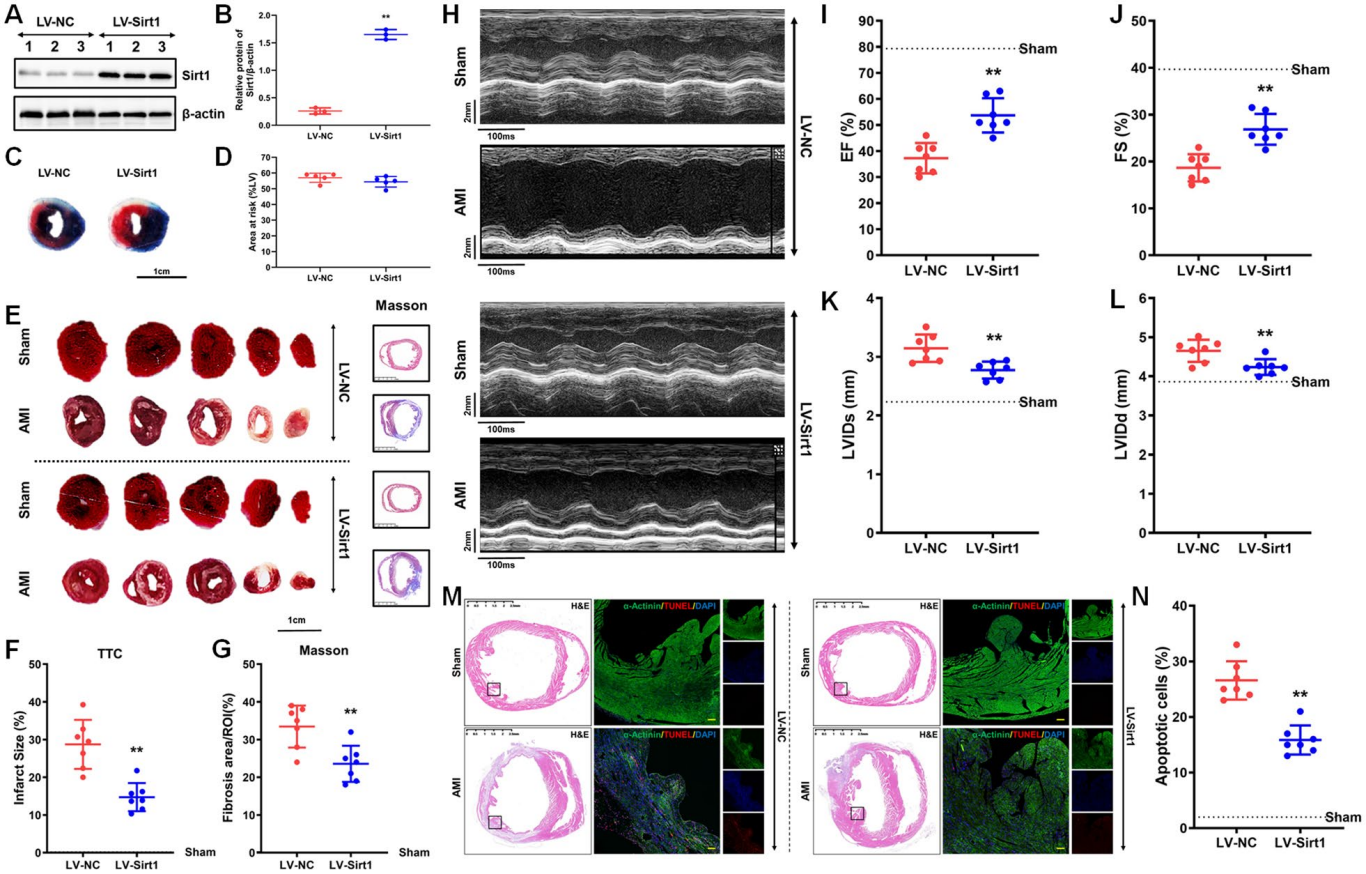
Overexpression of Sirt1 significantly reduced myocardial infarct size and myocyte apoptosis while improving cardiac function. **A**, **B** show the efficiency of Sirt1 overexpression via lentiviral transduction, confirmed by Western blot analysis (n = 3; the LV-NC group served as the control, with β-actin as the internal standard). **C**, **D** The area at risk in cardiac tissues subjected to LV-NC and LV-Sirt1 injections in the acute myocardial infarction (AMI) model was assessed using Evans blue staining (n = 5). **E**, **F** depict the quantification of myocardial infarct size in mice on day 7 post-MI using TTC staining, where non-infarcted myocardium is shown in red and infarcted myocardium in white. Fibrotic changes were analyzed in panels (**E** and **G**) using Masson staining, with integral optical density measurements recorded. ROI (Region of Interest) indicates the cross-sectional area of left ventricle stained. Left ventricular ejection fraction (EF), fractional shortening (FS) values, left ventricular internal diameter at end-systole (LVIDs) and left ventricular internal diameter at end-diastole (LVIDd) in AMI mice were measured via ultrasonic cardiac imaging (UGC) as shown in panels (**H**–**L**) (n = 7; **P < 0.01 compared to negative control [LV-NC], one-way ANOVA). Panels **M**, **N** display the evaluation of cardiomyocyte apoptosis, performed by TUNEL staining seven days post-MI. The left panel includes H&E staining and the right panel includes TUNEL staining where nuclei are blue (DAPI), apoptotic cells are red (TUNEL), and cardiomyocytes are green (α-actinin). The counts of apoptotic cells (n = 7 per group) were aggregated from ten infarcted regions (magnification: × 200; **P < 0.01 versus negative control [LV-NC], Kruskal–Wallis test)

To investigate the role of Sirt1 in AMI, researchers utilized a Sirt1-overexpressing lentiviral vector, which was transduced into the infarcted regions of mice subjected to MI (Higuchi et al. 2009), forming the LV-Sirt1 experimental group. Simultaneously, a negative control lentiviral vector was administered to another cohort of MI mice, designated as the LV-NC control group. Subsequent evaluations included assessments of myocardial infarction severity, cardiac function, and the extent of myocardial cell apoptosis. Verification of Sirt1 overexpression in cardiomyocytes within the infarcted tissues was conducted through western blotting and immunofluorescence analyses, confirming robust Sirt1 expression in the LV-Sirt1 group (Fig. 6A and B, Supplementary Fig. 3). Seven days post-MI, TTC staining, standardized to the area at risk relative to the left ventricle (LV) size (Fig. 6C and D), demonstrated a significantly reduced infarct size in the LV-Sirt1 group compared to the LV-NC group (LV-NC: 28.71 ± 6.50% versus LV-Sirt1: 14.72 ± 3.74%, P < 0.01; Fig. 6E and F). Similarly, Masson staining supported these findings by showing that Sirt1 overexpression effectively suppressed fibrotic remodeling during the post-MI period (Fig. 6E and G). Cardiac echocardiography provided compelling evidence of the cardioprotective effects of Sirt1, demonstrating significant improvements in cardiac function in the LV-Sirt1 group compared to the LV-NC group (Fig. 6H). This was reflected by a higher ejection fraction (EF: 53.71 ± 6.58% vs. 37.29 ± 5.82%, P < 0.01, Fig. 6I) and enhanced fractional shortening (FS: 26.86 ± 3.29% vs. 18.64 ± 2.91%, P < 0.01, Fig. 6J). Furthermore, Sirt1 overexpression mitigated adverse structural remodeling caused by AMI, as evidenced by a reduction in left ventricular internal diameter during both systole (LVIDs: 2.77 ± 0.14 mm vs. 3.15 ± 0.23 mm, P < 0.01, Fig. 6K) and diastole (LVIDd: 4.24 ± 0.20 mm vs. 4.65 ± 0.28 mm, P < 0.01, Fig. 6L). These findings highlight the therapeutic potential of Sirt1 in preserving cardiac structure and function. To further explore the mechanistic relationship between Sirt1 and cardiomyocyte apoptosis, histological analyses using H&E and TUNEL staining were performed to quantify apoptotic cardiomyocytes (α-actinin-positive cells, green) within the infarcted tissues seven days after lentiviral transduction. The results revealed a significantly lower incidence of cardiomyocyte apoptosis in the LV-Sirt1 group compared to the LV-NC group (LV-NC: 26.57 ± 3.46% vs. LV-Sirt1: 15.86 ± 2.61%, P < 0.01; Fig. 6M and N). Survival curve and statistical analysis shows both groups maintained a high survival rate over the 7-day observation period (Log-rank test, P = 0.1517, Supplementary Fig. 4). Collectively, these findings offer compelling evidence that Sirt1 overexpression provides a protective effect in the context of myocardial infarction by reducing infarct size, alleviating cardiac fibrosis, improving overall cardiac function, and suppressing apoptotic pathways in cardiomyocytes.

## Discussion

Our findings reveal that AMI significantly reduces Sirt1 expression while increasing Hif-1α levels and the cleaved caspase-3 to caspase-3 ratio. In vitro hypoxia models replicated these effects, showing a time-dependent decline in Sirt1 and elevated Hif-1α and cleaved caspase-3 in cardiomyocytes. Both AMI and hypoxia were linked to reduced expression of Phd3, an upstream regulator of Hif-1α. Overexpression of Sirt1 restored Phd3 levels, subsequently lowering Hif-1α and cleaved caspase-3, thereby mitigating hypoxia-induced apoptosis. Conversely, Phd3 knockdown abolished the inhibitory effect of Sirt1 on Hif-1α, while Hif-1α knockdown enhanced Sirt1 expression. In vivo, Sirt1 overexpression in infarcted myocardial regions reduced infarct size, decreased apoptosis, and improved cardiac function. Collectively, these findings demonstrate that hypoxia-induced downregulation of Sirt1 following AMI suppresses Phd3 expression, increases Hif-1α, and promotes cardiomyocyte apoptosis, ultimately impairing cardiac performance.

AMI caused by the occlusion of coronary arteries, triggers a cascade of harmful events marked by extensive myocardial ischemia and hypoxia (Burke and Virmani 2007). This hypoxic environment plays a critical role in inducing cardiomyocyte apoptosis, which significantly contributes to adverse cardiac remodeling and subsequent cardiac dysfunction post-AMI (Corbalan et al. 2016; Yaoita et al. 2000). Hypoxia profoundly alters gene expression profiles, most notably leading to a marked upregulation of Hif-1α levels (Guillemin and Krasnow 1997; Lee et al. 2004; Liu et al. 2018). Hif-1α can have protective effects in the early stages of MI by promoting hypoxia tolerance and myocardial protection. However, its impact is dose-dependent. While moderate levels of Hif-1α may be beneficial, excessive expression can paradoxically exacerbate ischemic injury (Wu et al. 2019). Elevated Hif-1α expression in the days following AMI has been shown by our research and others to exacerbate cardiomyocyte apoptosis and impair cardiac function (Wu et al. 2019). Conversely, Sirt1, a NAD^+^-dependent class III histone deacetylase, is vital for maintaining cellular homeostasis and has been implicated in various cardioprotective mechanisms (Dali-Youcef et al. 2007; Finkel et al. 2009; Watroba et al. 2017). Recent studies have emphasized the role of Sirt1 in mitigating hypoxia-induced cardiomyocyte apoptosis; for example, resveratrol, a known Sirt1 activator, has been shown to inhibit apoptosis induced by hypoxia and doxorubicin, highlighting the protective role of Sirt1 in the context of AMI (Hu et al. 2017; Zhang et al. 2011). In our study, we observed a significant decrease in Sirt1 expression following hypoxia, both in vivo and in vitro, accompanied by increased levels of Hif-1α and cleaved caspase-3, a key apoptotic marker. These findings suggest that the balance between Sirt1 and Hif-1α is crucial in regulating hypoxia-induced cardiomyocyte apoptosis. Mechanistically, hypoxia has been shown to increase the NADH/NAD^+^ ratio, which inhibits Sirt1 activity (Zhang et al. 2015), potentially explaining the observed downregulation of Sirt1. Notably, Sirt1 expression was significantly restored seven days post-AMI, coinciding with the recovery phase and improved cardiac function (Pendergrass et al. 2011). In murine models, low to moderate cardiac-specific overexpression of Sirt1 has been shown to effectively alleviate age-related progression of cardiac hypertrophy, fibrosis, apoptosis, and decline in cardiac function (Alcendor et al. 2007). The moderate overexpression of Sirt1 in the infarcted myocardium led to a reduction in infarct size, a decrease in cardiomyocyte apoptosis, and a significant improvement in cardiac function (Alcendor, Gao, Zhai, Zablocki, Holle, Yu, Tian, Wagner, Vatner and Sadoshima 2007). On the other hand, we also found that AMI leads to a decrease in Sirt1 expression while concurrently increasing Sirt7 expression, a pattern consistent with previous studies (Araki et al. 2015; Hsu et al. 2010). These results suggest that the compensatory upregulation of Sirt7 may serve to counterbalance the deleterious effects of diminished Sirt1 activity. AMI induces a marked decrease in Sirt1 expression, which correlates with increased p53 acetylation and activation of apoptotic pathways, ultimately leading to cardiomyocyte death (Mu et al. 2014). Conversely, the concurrent upregulation of Sirt7 appears to exert a cardioprotective effect by deacetylating p53 and attenuating its pro-apoptotic signaling during hypoxic stresss (Sun et al. 2018; Zhan et al. 2017). Furthermore, Sirt7 indirectly promotes Sirt1 activity by repressing the transcription of DBC1, a known Sirt1 inhibitor, thereby enhancing downstream survival pathways such as AKT signaling (Li et al. 2019). These findings reveal a pivotal Sirt1-Sirt7 axis that modulates the balance between cardiomyocyte survival and apoptosis in ischemic conditions. Collectively, these findings suggest that increasing Sirt1 levels alleviates myocardial imbalances caused by hypoxia and provides cardioprotective effects following AMI.

Our study elucidates a novel mechanism through which Sirt1 exerts its cardioprotective effects, primarily by regulating Phd3, a critical modulator of Hif-1α stability. We demonstrate that Sirt1 overexpression leads to an upregulation of Phd3, which subsequently inhibits Hif-1α expression, thereby reducing cardiomyocyte apoptosis. This effect was definitively abolished by Phd3 knockdown, confirming that Phd3 acts as an essential mediator within the Sirt1-Hif-1α signaling pathway. Phd proteins, including Phd3, regulate Hif-1α levels via post-translational hydroxylation, promoting its proteasomal degradation, as supported by prior literature (Taylor 2001). Moreover, the relationship between Sirt1 and Hif-1α is characterized as dynamic and reciprocal, underscored by the observation that Hif-1α knockdown appears to enhance Sirt1 expression, suggesting a mutually reinforcing regulatory loop. The role of Phd3 in cardiac pathophysiology exhibits considerable complexity and context dependency. While Phd3 is typically expressed at high levels in both the heart and placenta (Fialho et al. 2019; Myllyharju and Koivunen 2013), existing literature presents seemingly contradictory findings regarding Phd3 modulation in AMI models. Specifically, Phd3 knockout has been linked to enhanced angiogenesis and improved cardiac function (Oriowo, et al. 2014), whereas its overexpression has been associated with increased cellular survival under hypoxic conditions (Hogel et al. 2011). Notably, our findings reveal a temporal decline in Phd3 expression within infarcted myocardial tissue and hypoxic cardiomyocytes, indicating that Phd3’s role may depend on the injury stage and specific cellular environment. It has been reported that SIRT1 in neurons deacetylates and inhibit pyruvate kinase M2 (PKM2), a Phd3-stimulated coactivator for Hif-1α (Luo et al. 2011). Therefore, in normoxic condition, Sirt1 in cardiomyocytes may upregulate Phd3 via suppression of Hif-1α expression. In addition, Sirt1 regulates the expression of apoptosis-related genes by promoting Stat1 phosphorylation (Yang et al. 2018), and phosphorylated Stat1 (p-Stat1) is known as a Hif-1α-independent transcriptional regulator of Phd3 expression (Gerber et al. 2009), indicating a potential mechanism that in cardiomyocytes Sirt1 increases Phd3 expression via p-Stat1. Furthermore, we observed difference of Phd3 expression and subcellular distribution under normoxic and hypoxic conditions, suggesting potential post-translational modifications. Future studies are needed to investigate the specific modifying role of Sirt1 on Phd3, clarifying whether this modification impacts Phd3 localization, degradation, and function. Despite extensive research on Phd3 in cancer biology (Chu et al. 2019; Xia et al. 2020), its role in cardiomyocytes remains insufficiently explored. Thus, our study offers groundbreaking insights into the cardiomyocyte-specific functions of Phd3, particularly in its regulation of Hif-1α under hypoxic conditions downstream of Sirt1.

## Conclusion

In conclusion, our study reveals a previously unrecognized mechanism by which Sirt1 mitigates hypoxia-induced apoptosis in cardiomyocytes through modulation of the Phd3/Hif-1α signaling pathway. By showing that Sirt1 promotes the upregulation of Phd3, thereby suppressing Hif-1α activation and subsequent apoptotic processes, we provide significant insights into the molecular mechanisms that support cardiomyocyte survival in hypoxic conditions. These findings not only enhance our understanding of the functional role of Sirt1 in AMI but also highlight the Sirt1/Phd3/Hif-1α signaling axis as a promising therapeutic target for improving cardiac function following myocardial injury.

## Supplementary Information

**Additional file 1 : **Figure S1. Bioinformatics analysis of cardiomyocytes exposed to normoxia or hypoxia.Heatmap of genes differentially expressed in cardiomyocytes from neonatal mice exposed to either normoxia or hypoxia condition. Log2 fold changeof the expression of sirtuin family genes between the hypoxic and normoxic groups. The color of the bars indicates the significance level of changes

**Additional file 2 : **Figure S2. The expression and localization of Phd3 in the infarction region of AMI mice

**Additional file 3 :** Figure S3. The expression and location of Sirt1 in cardiomyocytes in the infarction region of AMI mice after LV-NC or LV-Sirt1 injection

**Additional file 4 : **Figure S4. Survival curves of control or Sirt1-overexpressing mice. The figure shows the survival curves of LV-NCand LV-Sirt1mice. The X-axis represents time, and the Y-axis represents survival rate. Statistical analysis using the Log-rank test revealed no significant difference in survival rates between the two groups. Both groups maintained a high survival rate over the 7-day observation period, with a slight decrease in the LV-NC group around day 4

**Additional file 5.**

## Data Availability

The datasets used and/or analyzed during the current study are available from the corresponding author on reasonable request.

## Abbreviations

Sirt1: Sirtuin1
Hif-1α: Hypoxia-inducible factor 1α
Phd3: Prolyl hydroxylase 3
AMI: Acute myocardial infarction
LAD: Left anterior descending
MI: Myocardial infarction
TTC: 2,3,5-Triphenyl tetrazolium chloride
Echo: Echocardiography
