# Supplementary figures and images for: Sirtuin1 mitigates hypoxia-induced cardiomyocyte apoptosis in myocardial infarction via PHD3/HIF-1α
Source: Mol Med. 2025 Mar 14;31:100. doi: 10.1186/s10020-025-01155-z (PMC11909899; doi:10.1186/s10020-025-01155-z)

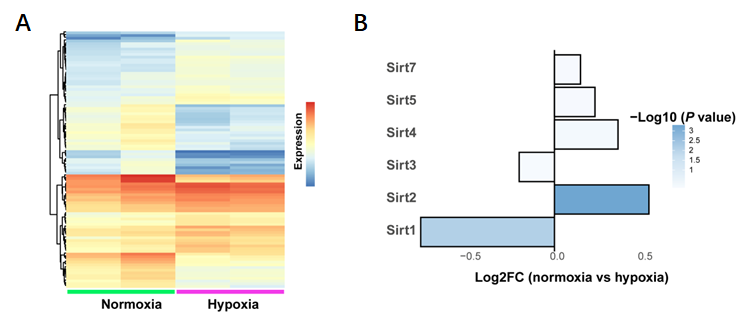

Supplement: Supplementary file 1 — Additional file 1 : Figure S1. Bioinformatics analysis of cardiomyocytes exposed to normoxia or hypoxia.Heatmap of genes differentially expressed in cardiomyocytes from neonatal mice exposed to either normoxia or hypoxia condition. Log2 fold changeof the expression of sirtuin family genes between the hypoxic and normoxic groups. The color of the bars indicates the significance level of changes [file 10020_2025_1155_MOESM1_ESM.tif]

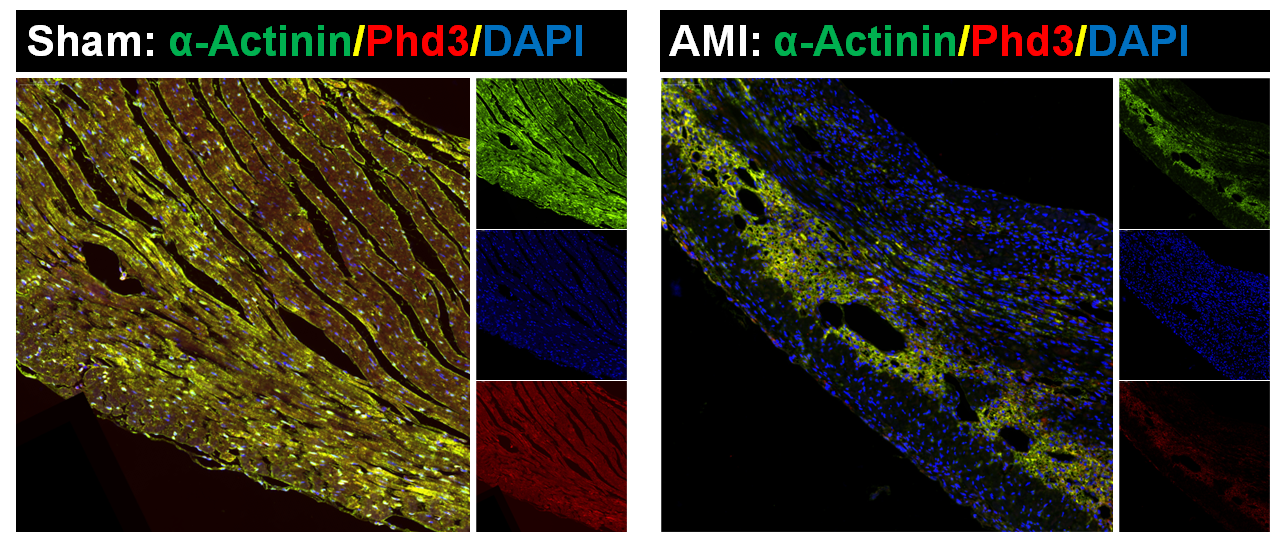

Supplement: Supplementary file 2 — Additional file 2 : Figure S2. The expression and localization of Phd3 in the infarction region of AMI mice [file 10020_2025_1155_MOESM2_ESM.tif]

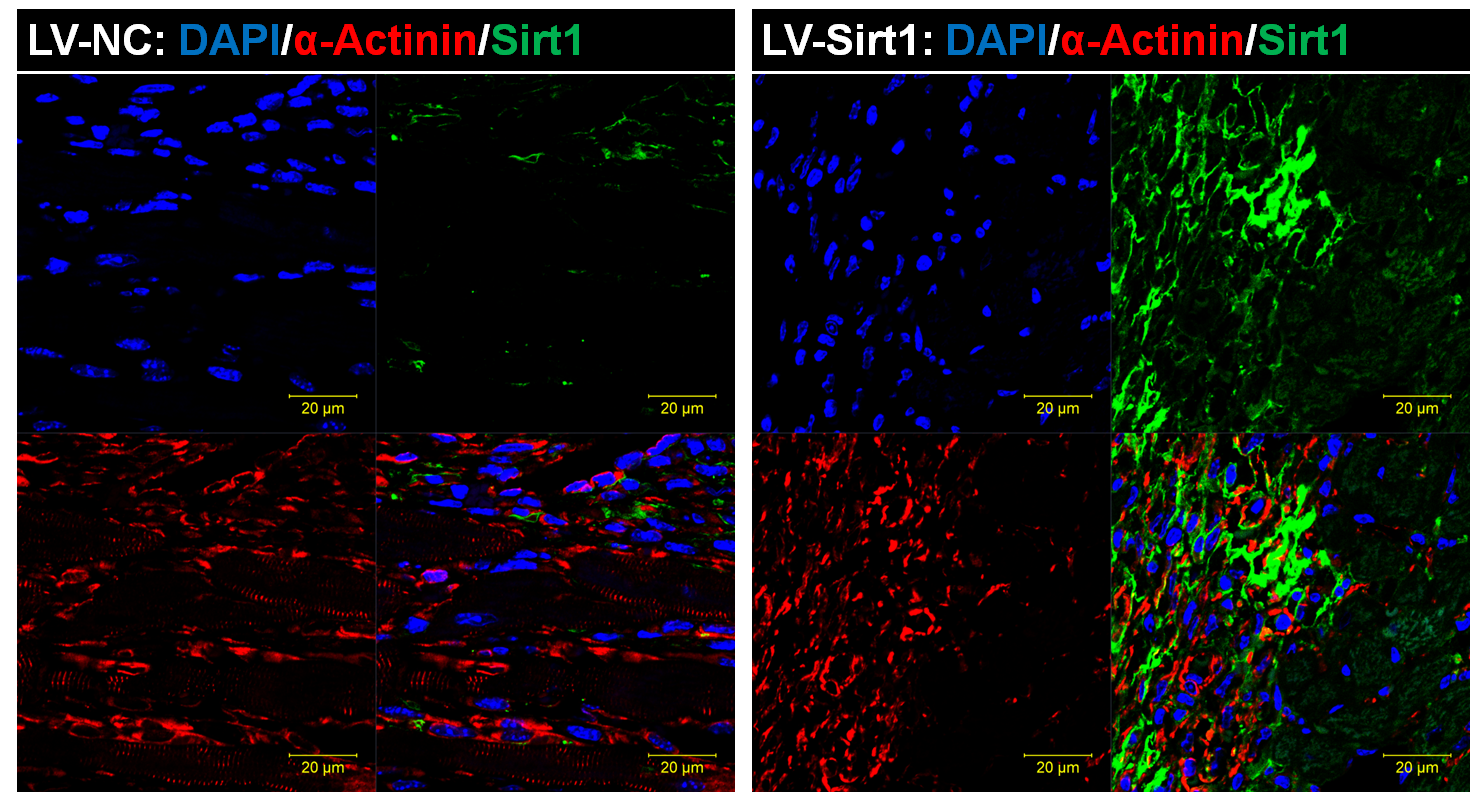

Supplement: Supplementary file 3 — Additional file 3 : Figure S3. The expression and location of Sirt1 in cardiomyocytes in the infarction region of AMI mice after LV-NC or LV-Sirt1 injection [file 10020_2025_1155_MOESM3_ESM.tif]

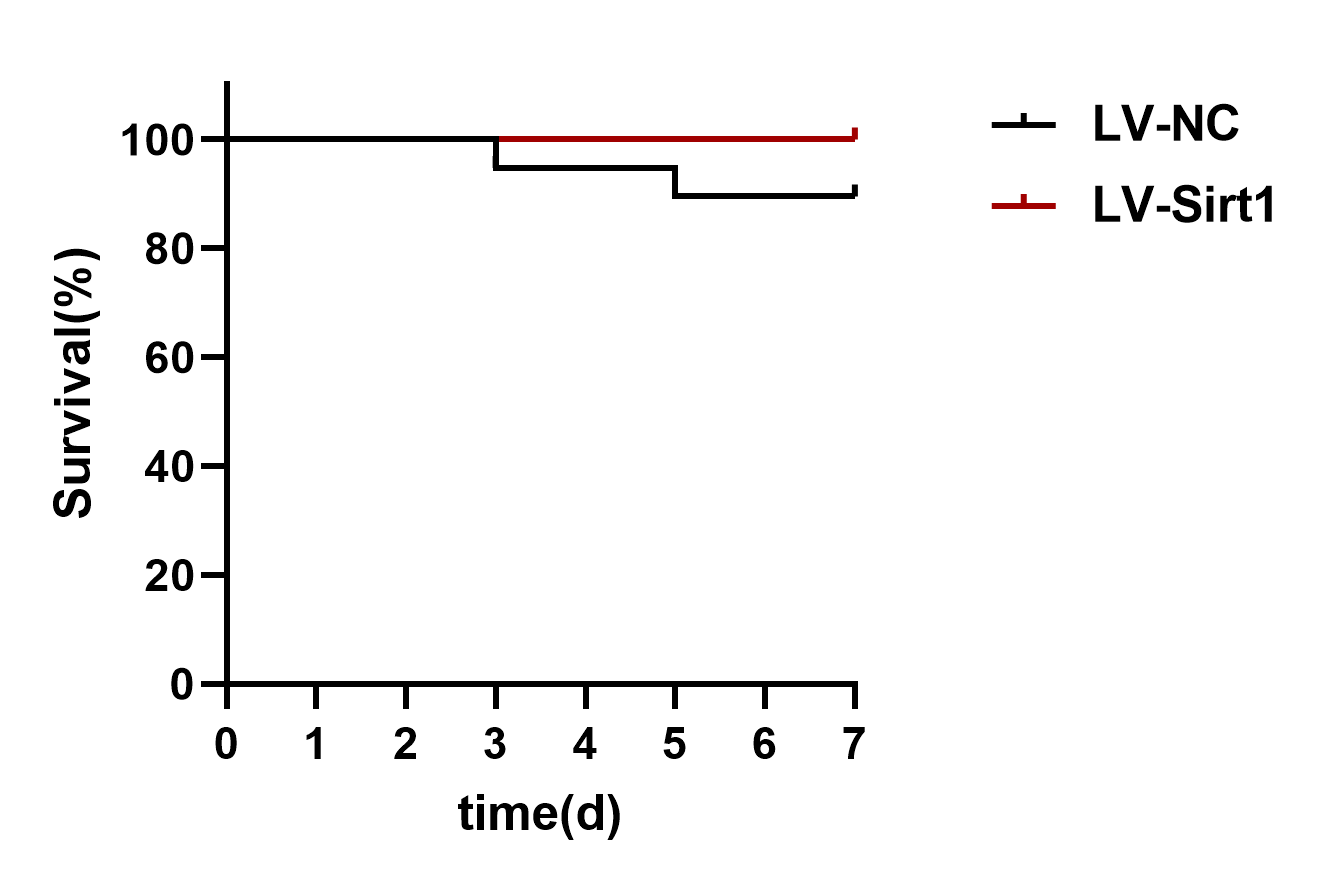

Supplement: Supplementary file 4 — Additional file 4 : Figure S4. Survival curves of control or Sirt1-overexpressing mice. The figure shows the survival curves of LV-NCand LV-Sirt1mice. The X-axis represents time, and the Y-axis represents survival rate. Statistical analysis using the Log-rank test revealed no significant difference in survival rates between the two groups. Both groups maintained a high survival rate over the 7-day observation period, with a slight decrease in the LV-NC group around day 4 [file 10020_2025_1155_MOESM4_ESM.tif]
