# Supplementary material for: Sirtuin1 mitigates hypoxia-induced cardiomyocyte apoptosis in myocardial infarction via PHD3/HIF-1α
Source: Mol Med. 2025 Mar 14;31:100. doi: 10.1186/s10020-025-01155-z (PMC11909899; doi:10.1186/s10020-025-01155-z)
Supplement: Supplementary file 5 — Additional file 5. [file 10020_2025_1155_MOESM5_ESM.docx]

**Table S1. Primer sequences**.

| Sirt1 | Forward primer: TATCTATG CTCGCCTTGC  Reverse primer: GAATGGTCT TGGGTCTTT |
| --- | --- |
| Sirt2 | Forward primer: CCTTCTTTGCCCTTGCC  Reverse primer: CCAGCCCATCGTGTATTCT |
| Sirt3 | Forward primer: CCCCGACTGCTCATCAA  Reverse primer: CCACCAGCCTTTCCACA |
| Sirt4 | Forward primer: CGGTGAACCCAGACAAGG  Reverse primer: CAGGCAAGCCAAATCGT |
| Sirt5 | Forward primer: AACGCCAAGCACATAGCC  Reverse primer: TGCCCTGGTCACGAAGC |
| Sirt6 | Forward primer: TAGAGGAATGTCCCAAGTGTAAG  Reverse primer: TCATCAGCGAGCATCAGG |
| Sirt7 | Forward primer: GCCAGGCACTTGGTTGTC  Reverse primer: CTCCGCTTCGCTTAGGTC |
| Phd3 | Forward primer: GGCCATCAGCTTCCTCCTG  Reverse primer: GGTGATGCAGCGACCATCA |
| β-actin | Forward primer: CTGTCCCTGTATGCCTCTG  Reverse primer: ATGTCACGCACGATTTCC |
